# Application of New COF Materials in Secondary Battery Anode Materials

**DOI:** 10.3390/molecules28165953

**Published:** 2023-08-08

**Authors:** Miao Jia, Lixin Zhang, Qiong Yuan

**Affiliations:** 1 College of Chemistry and Materials Engineering, Beijing Technology and Business University, Beijing 100048, China; zhanglx0816@163.com; 2 College of Chemical and Food, Zhengzhou University of Technology, Zhengzhou 450044, China; 13716673969@163.com

**Keywords:** covalent organic framework, lithium-ion battery, sodium-ion battery, potassium-ion battery, energy storage mechanism

## Abstract

Covalent organic framework materials (COFs), as a new type of organic porous material, not only have the characteristics of flexible structure, abundant resources, environmental friendliness, etc., but also have the characteristics of a regular structure and uniform pore channels, so they have broad application prospects in secondary batteries. Their functional group structure, type, and number of active sites play a crucial role in the performance of different kinds of batteries. Therefore, this article starts from these aspects, summarizes the application and research progress of the COF anode materials used in lithium-ion batteries, sodium-ion batteries, and potassium-ion batteries in recent years, discusses the energy storage mechanism of COF materials, and expounds the application prospects of COF electrodes in the field of energy storage.

## 1. Introduction

With the gradual depletion of fossil energy sources, such as oil and natural gas, and demand for reductions in carbon dioxide emissions, the development and utilization of new energy and renewable clean energy storage technology have become an urgent problem to solve, as well as a topic of increasing concern [1,2,3]. Electrochemical energy storage, inductor energy storage, and gravitational potential energy storage are the main energy storage technologies currently developed. Because of its efficient and flexible application, electrochemical energy storage enjoys an increasingly important role in energy storage systems [1,4]. At present, electrochemical energy storage includes battery energy storage and capacitor energy storage. Ordinary batteries include lithium-ion batteries, sodium-ion batteries, potassium-ion batteries, supercapacitors, etc. Secondary batteries are usually composed of a cathode material, anode material, electrolyte, diaphragm, and other parts, among which the anode material plays a very important role in the performance of the battery.

Generally speaking, the electrode materials of secondary batteries include carbon materials, alloy materials, metal oxide/sulfur/selenide materials, and polymer materials. Carbon materials have advanced technology and low cost but low specific capacity and poor safety [5]. The alloy material has high capacity but large volume change, causing poor circulation performance [6]; metal oxide/sulfur/selenide materials have high specific capacity but poor conductivity, and they are prone to volume expansion [7]. Among various anode electrode materials, some organic compounds with rich conjugation systems and lone pair electrons can undergo reversible oxidation reduction reactions without relying on transition metal element resources; meanwhile, the structure and performance are easy to design and control, so they have certain potential.

Among polymer anodes, covalent organic frameworks (COFs) are new crystalline porous materials composed of light organic molecular elements (H, B, C, N, O, etc.) and connected by full covalent bonds to form a two-dimensional or three-dimensional network structure, with excellent structural controllability and functional adjustability. Compared with traditional metal organic framework (MOF) materials, COFs have a more orderly pore structure and higher stability. In addition, the advantages, such as low skeleton density, high porosity, and pore structure, give COFs broad application prospects in gas storage, adsorption and separation, photoelectric conversion, heterogeneous catalysis, and other fields [8,9].

**Figure 1:**
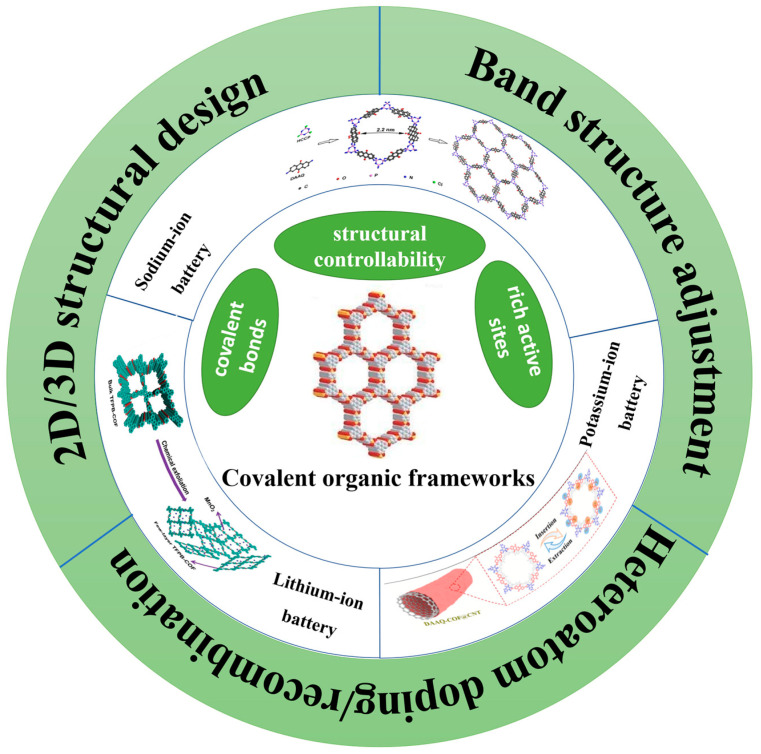
Application of COF materials in anode of secondary-ion batteries [10,11,12].

In recent years, COFs have been gradually used in various secondary battery anode materials, including lithium-ion batteries, sodium-ion batteries, potassium-ion batteries, etc. (Figure 1) [10,11,12]. Compared with traditional organic composite electrode materials, the regular network structure of COFs can obtain richer redox active sites, which is conducive to ion/electron transmission. Therefore, COFs, as anode materials of secondary batteries, have better electrochemical properties [13]. In addition to alkali-ion battery anode materials, COFs are also promising for multivalent ion batteries, like Mg-ion, Ca-ion, and Zn-ion batteries [14,15,16,17]. Due to adjustable pore size, regular channel structure, and rich redox active sites, COFs may be potential electrode materials for multivalent ion batteries. Tunable and stable porous structure is conducive to Mg^2+^, Ca^2+^, and Zn^2+^ storage. Most efforts so far have been made on COFs directly as cathode materials for multivalent ion batteries, and less research is about the application of COFs on anodes. The application of COFs on anodes revolves in modifying the Mg, Ca, or Zn anode as protective layers. Zhao et al. prepared a COFs@Zn anode (TpPa-SO_3_H@Zn-foil) for zinc-ion batteries, which effectively promoted the uniform deposition of zinc ions and inhibited dendrite growth [18]. Zhang et al. prepared TB-COF as an anode for calcium-ion batteries, demonstrating the high reversible capacity of 253 mA h g^−1^ at 1.0 A g^−1^ [19]. Apparently, COFs play an important role in the energy storage of multivalent ions, the same as for alkali-ion batteries. In addition, aqueous batteries have shown certain progress. Lin et al. [20] prepared a kind of anode material containing pyrozine (C=N) and phenylimino (-NH-) groups as a water system battery. The strong covalent bond of COF and the hydrogen bond network between -NH- and water molecules jointly improved the acid–base resistance and accelerated the ion transmission. Therefore, the COF electrode has excellent capacity and cycle stability. At present, the research of COFs used for new types of secondary battery is in the initial stage, and there will be more research on COFs in the future. Meanwhile, in supercapacitors, COF materials have relatively sufficient research, but the transmission speed of supercapacitors through electrodes is slower, so their power density is relatively low [21,22].

Currently, COF anode materials face inherent challenges, such as poor electronic conductivity, potential solubility in electrolytes, and low volume capacity. Therefore, this paper summarizes the corresponding solutions and the application of COFs and their composites in the field of lithium/sodium/potassium secondary battery anodes, including the latest achievements and the reaction mechanism of COFs in the energy storage process. Finally, the challenges faced by COFs in the field of secondary batteries are discussed, and future developments are prospected.

## 2. Application of COF Materials in the Lithium-Ion Battery Anode

### 2.1. COF Anode

Lithium-ion batteries (LIBs) have the advantages of high voltage and light weight. The battery voltage is usually about 3.6 V, which is equivalent to the series voltage of 2–4 Ni-MH batteries; meanwhile, lithium-ion batteries have high energy density. The density of lithium-ion batteries commonly used at present can reach 180 Wh kg^−1^, which is 5–7 times that of lead-acid batteries. In addition, lithium-ion batteries have long service life, low self-discharge, and no memory effect [23,24]. At present, in portable electronic devices, new energy vehicles, and other technologies, lithium-ion batteries have gradually shown their advantages. With the advent of the era of the smart grid, lithium-ion batteries have also been rapidly developed on a large scale [25,26].

As an anode of lithium-ion batteries, the following requirements should be met: when lithium ions are introduced into the anode matrix, the oxidation reduction potential should be as low as possible, close to the metallic lithium potential to increase the input voltage of the battery; during the intercalation/deintercalation process, the main structure change in the anode electrode is insignificant or does not change at all; the oxidation reduction potential should also change as little as possible, so that the battery voltage will not change significantly and can maintain a relatively stable charge/discharge voltage; the inserted compound should have excellent electronic/ionic conductivity, which can reduce polarization and can be charged and discharged under large current density; the main materials should have a valuable surface structure and be able to form a good SEI with liquid electrolytes; lithium ion has a large diffusion coefficient in the base material, which is convenient for fast charging and discharging; from a practical point of view, the material should be economical and environmentally friendly [27].

At present, two-dimensional COFs are relatively widely used in lithium-ion batteries, which are usually composed of a planar network structure connected by rigid building units and stacked by π–π interaction between layers. Theoretically, the overlap of π–π electron clouds between layers and the conjugated connection within layers enable the carrier to transfer rapidly in the COF skeleton [28]. At present, many kinds of two-dimensional COF materials have been prepared and have obtained excellent performance in lithium-ion battery anodes.

**Figure 2:**
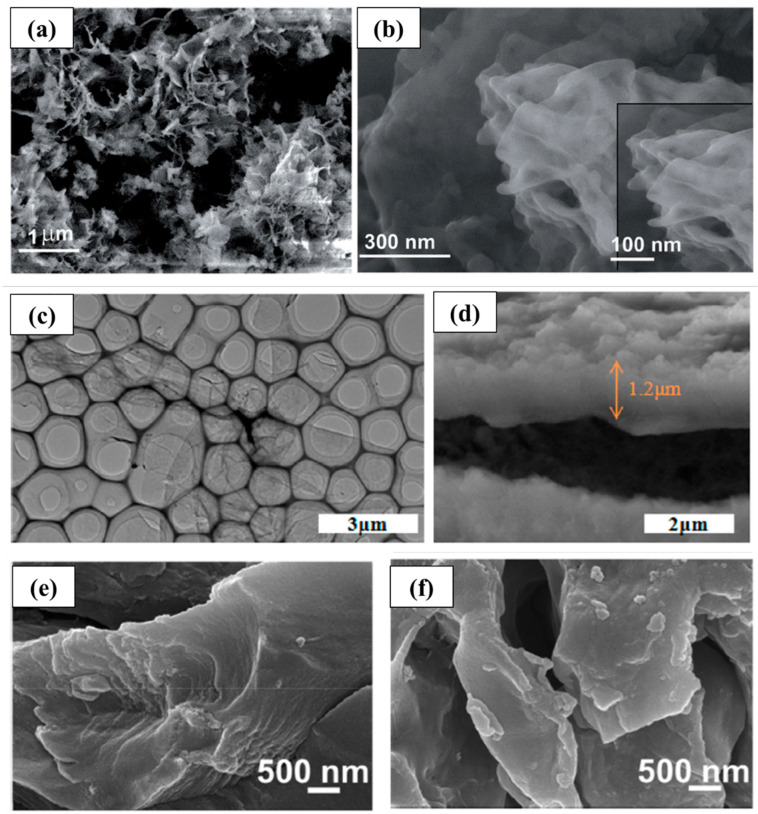
Research on 2D COF in lithium-ion batteries. (**a**,**b**) FE-SEM and higher-resolution FE-SEM of very thin nanosheets (CON) [29]; (**c**,**d**) TEM image of as-exfoliated nanosheets (TThPP) [30]; (**e**,**f**) SEM images of DCB-COF-450 and DCB-COF-500 nanosheets [33].

Haldar et al. [29] synthesized self-assembled covalent organic skeleton nanosheets (Figure 2a,b) using the solvothermal method. This two-dimensional structure can shorten the transport path and is conducive to the rapid intercalation/deintercalation of Li^+^, so it has high reversibility and cycle stability. This study proved that COFs are very suitable for ion insertion in the form of stripping. Yang et al. [30] grew highly conductive two-dimensional COF polyporphyrin (TThPP) connected by 4-thiophenephenyl on the surface of copper foil through in situ chemical oxidation polymerization. As the anode of lithium-ion batteries, the regular arrangement of two-dimensional polyporphyrin nanosheets (Figure 2c,d) facilitates the flow of charge carriers on the COF framework, thereby improving electronic conductivity. Simultaneously, the structure endows the composite with more active sites and large specific surface area, which can promote the rapid diffusion and effectively shorten the transmission path of Li^+^. Zhao et al. [31] synthesized a layered porous covalent organic framework with C=N and C=O double redox-active centers through the condensation reaction of 2,6-diaminoanthraquinone (DAAQ) and 1,3,5- benzenetricarboxaldehyde (TB). This 2D DAAQ-COF plane is stacked and aggregated through strong π–π interactions, forming a layered structure; meanwhile, the introduction of the C=O helps to increase the active sites and improve the theoretical capacity. Thus, DAAQ-COF exhibits excellent performance and continuous “activation” behavior. After 500 cycles under 1 A g^−1^, the discharge capacity is up to 787 mA h g^−1^. Wu et al. [32] designed and prepared a redox-active PA-TA COF with super-large interlayer spacing, which had a non-planar tetrahedral chair conformational piperazine unit. This unique structure gives it a bigger interlayer distance. Compared with commercial graphite and two-dimensional COF anode materials with dense aromatic stacking, the ultra-large interlayer spacing of PA-TA COF can effectively promote the intercalation/deintercalation of Li^+^ with fast transport kinetics, thereby increasing the rate capacity. After 400 cycles at 1.0 A g^−1^, the specific capacity of the composite material still reaches 543 mA h g^−1^, while at a high current density of 5.0 A g^−1^, its specific capacity can still reach 207 mA h g^−1^. Cai et al. [33] synthesized a π-conjugated covalent organic framework (DCB-COF) based on a triazine (DCB) structure (Figure 2e,f). As the anode material of the lithium-ion battery, the active sites for lithium storage of DCB-COF are attributed to its C-N functional group and the π-conjugated benzene ring, which cause strong cycle stability, high energy storage, and power supply capacity.

**Figure 3:**
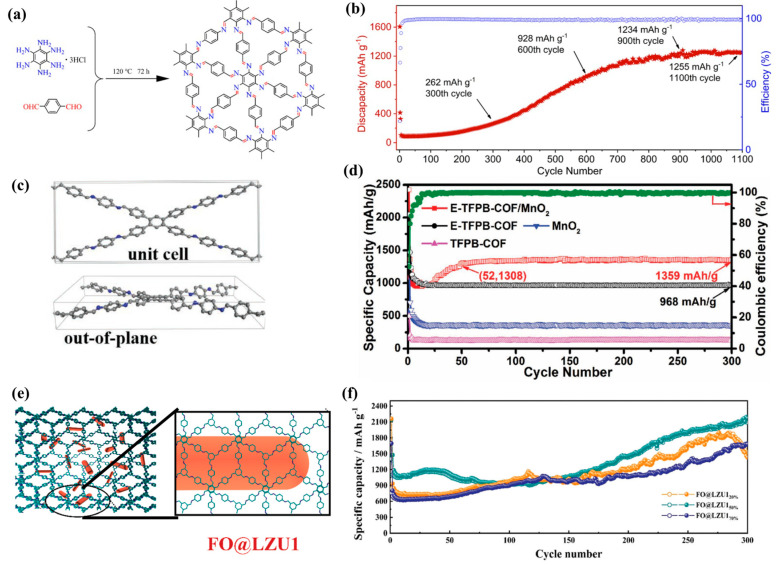
Structure and properties of some two-dimensional COFs. (**a**) Synthesis of HAB-COF; (**b**) the electrochemical performance of the HAB-COF under 1 A g^−1^ (first three cycles are under 0.1 A g^−1^) [34]; (**c**) characterization of layered TFPB-COF unit cell, (**d**) electrochemical performances of E-TFPB-COF/MnO_2_ products at 0.1 A g^−1^ [10]; (**e**) characterization of FO@LZU1, (**f**) cycle performance of FO@LZU products [35].

In addition to carbonyl (C=O), triazine structure, and other functional groups, the imine group (C=N) is also a typical active site for COFs in Li-ion storage. Zhao et al. [34] synthesized a new triangular topological covalent organic framework (HAB-COF) based on hexaaminobenzene. Due to its conjugated structure and high-density imine (C=N) groups (Figure 3a), it effectively promotes electron transport and Li^+^ intercalation/deintercalation. At the same time, HAB-COF undergoes an activation stage during the electrochemical cycling process, and the specific capacity reaches 1255 mA h g^−1^ after 1100 cycles at 1 A g^−1^ (Figure 3b), far exceeding the theoretical specific capacity based solely on C=N group materials. This may be due to the cyclic synergistic effect of the stable activated C=C group in benzene on HAB and Td monomers. Chen et al. [10] designed a novel imine-based COF (TFPB COF) through the Schiff base reaction and chemically peeled it off into multi-layer nanosheets. Its structure is similar to that of graphene (Figure 3c), with an ordered mesoporous structure and large specific surface area, which can effectively promote ion/electron diffusion, so it has excellent lithium storage performance. In addition, MnO_2_ can act as a spacer between layers, effectively preventing the aggregation of composite materials during cycling, thus also possessing long-term cycling stability. The E-TFPB-COF/MnO_2_ electrode showed a large reversible capacity of 1359 mA h g^−1^ after 300 cycles, without the need for prolonged cycling activation (Figure 3d). Therefore, the design of composite materials has a significant impact on improving battery performance. Long et al. [35] synthesized Fe_2_O_3_@COF-LZU1 (FO@LZU1) composites. The existence of COF-LZU1 increases the additional active site for lithium storage, as well as providing a unique heterostructure. A large number of C=N groups and benzene rings exist in imine-based COFs, providing a rich active center for lithium storage through electrochemical reaction (Figure 3e). In addition, its ordered pores promote the permeation of electrolytes in each layer, and the rigid skeleton can improve the structural stability and limit the volume expansion of Fe_2_O_3_. Therefore, FO@LZU1_50%_ showed high initial capacity and long-term cycling performance, with a capacity of 2171 mA h g^−1^ after 300 cycles at 0.1 A g^−1^ (Figure 3f).

**Figure 4:**
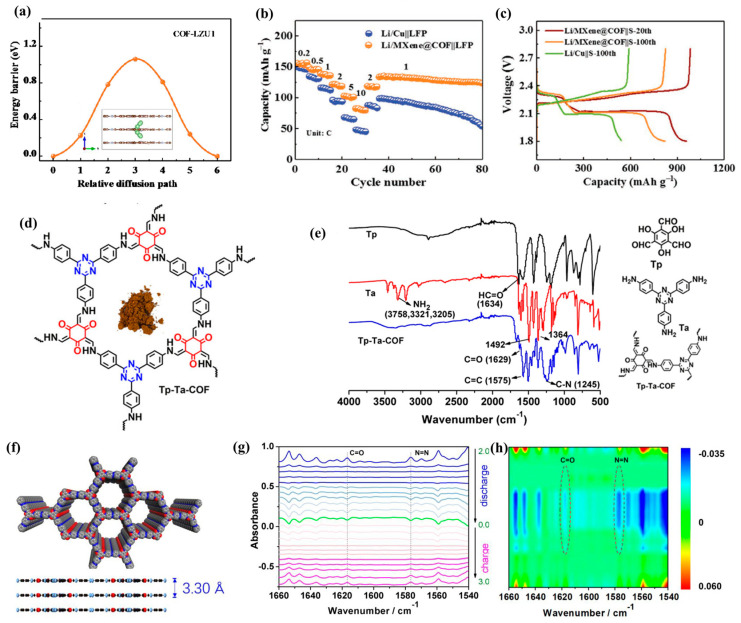
(**a**) Diffusion kinetics of Li ions in pristine COF-LZU1; (**b**) rate performance (**c**) discharge/charge curves of MXene@COF products in full batteries [38]; (**d**) structural characterization of Tp-Ta-COF AA stacking mode, (**e**) FT-IR spectra of starting materials of Tp, Ta, and as-prepared Tp-Ta-COF [39]; (**f**) schematic diagram of Tp-Azo-COF preparation, (**g**) in situ FT-IR spectra changes in electrode samples under different discharge states, (**h**) contour and response surface analysis corresponding to discharge process [40].

In addition, COFs are often compounded with graphene, carbon nanotubes, MXene, and other materials. To improve the capacity and long cycle stability of COF materials, Wang et al. [36] modified dioxin-chain COF with graphene quantum dots. This composite material introduced graphene quantum dots rich in carboxyl groups, thus changing the surface structure of COF, exposing more active sites, and promoting the transport dynamics of lithium ions. Yang et al. [37] synthesized the COF@CNT composite material. The introduction of carbon nanotubes helped COF shorten the ion/electron transport path, expose more active sites, and improve the conductivity. Therefore, the composite material has excellent lithium storage performance, with a specific capacity of approximately 570 mA h g^−1^ after 100 cycles under 0.1 A g^−1^. Guo et al. [38] compounded COF materials with MXene. The aminated 2D MXene provides reactive sites for the interface-initiated imine bond formation, which is conducive to the nucleation of COF and the growth towards mixed heterostructures on the MXene layer. The heterostructure brought by two two-dimensional materials significantly improves the lithium storage and charge transfer performance of the composite material (Figure 4a–c).

It can be seen from the above that to improve the electrochemical properties of COF materials, it is important to increase the active sites of composite materials. Therefore, a variety of COFs with double-active groups have also been developed. Zhao et al. [39] prepared Tp-Ta-COF containing C=N and C=O double-active centers (Figure 4d). The diffusion kinetics was significantly enhanced, and the resistance and migration barrier were reduced, resulting in excellent lithium storage performance. In situ FT-IR and Raman (Figure 4e) combined with XPS and DFT calculation further prove that the interaction between the redox active site and lithium ions is highly reversible. Therefore, this composite material can deliver a high reversible capacity of 413 mA h g^−1^ under 200 mA g^−1^. Zhao et al. [40] designed a covalent organic framework (Tp-Azo-COF) with N=N and C=O (Figure 3f). The active sites were characterized via in situ FT-IR (Figure 4g,h). Such composite materials have a large surface area and lithium-ion transport path, so they have high electrochemical kinetics and structural stability. Tong et al. [13] developed a C=N and N=N double-active-center COF. After 100 cycles at 0.1 A g^−1^, the practical capacity of the COF electrode could achieve 433 mA h g^−1^.

### 2.2. The Lithium Storage Mechanism of COF Materials

The types of COF materials are complex, so their mechanisms of action in lithium-ion batteries are also different, but they still follow the basic “Rocking Chair Battery” principle of lithium-ion batteries. Energy storage and release in lithium-ion batteries are achieved by the movement of lithium ions between two electrodes through electrolytes under cyclic voltage. During the charging process, lithium ions will be released from the grid of the positive electrode material, thus improving the electrode potential of the positive electrode. At the same time, lithium ions move to the surface of the negative electrode together with the electrolyte and intercalate into the negative active material, resulting in a decrease in the potential of the negative electrode and an increase in the voltage difference between the positive and negative electrode, which realizes the charging of the lithium-ion battery; when discharging, the situation is exactly the opposite [41].

**Figure 5:**
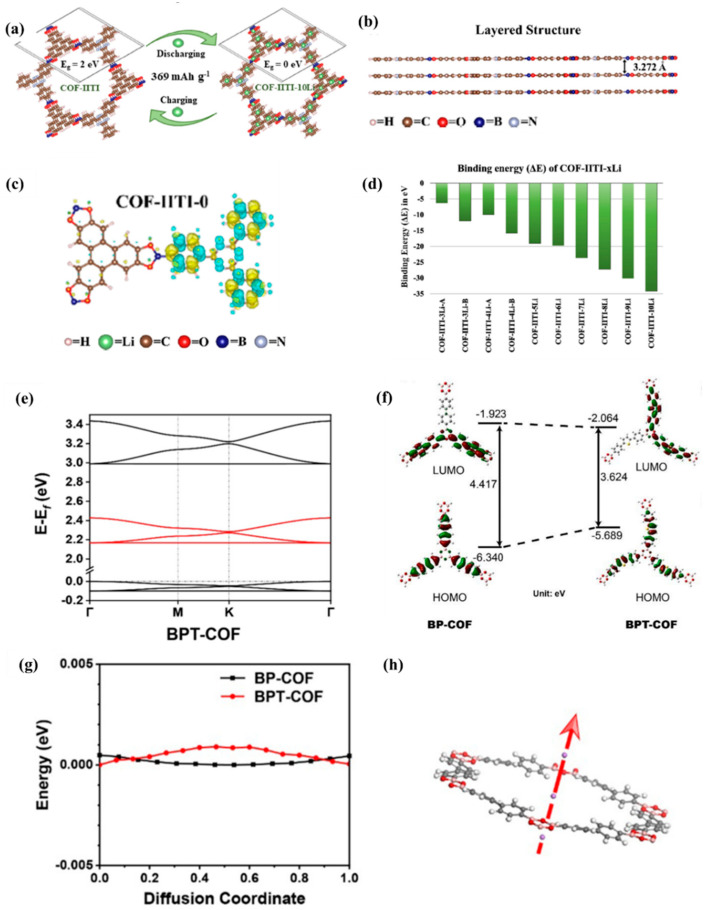
(**a**) Schematic diagram of Li storage and charge discharge process in lithium-ion battery electrodes using COF-IITI-0 as anode, (**b**) equilibrium layered structure of COF-IITI-0, (**c**) electron density of COF-IITI-0, (**d**) predicted binding energies (ΔE) of Li embedded in COF [42]. (**e**) Band structure of BPT-COF; (**f**) the HOMO/LUMO and energy level of BP-COF (**left**) and BPT-COF (**right**) molecules, (**g**) relative energy of cell when Li-ions pass through COF pores, (**h**) graphical representation of Li ions passing through COF pores [43].

Sinha et al. [42] explored the intercalation mechanism of lithium ions in COF materials using first-principles calculations. As shown in Figure 5a–d, structural parameter studies reveal that, due to the interatomic interaction between the COF layer and the embedded lithium atom, the embedding of lithium atoms will affect the lattice constant, structural parameters, equilibrium bond length, and interlayer distance. In addition, the cohesive energy of COF material is relatively high, about 7.34 eV/electron, which is very close to that of graphene, so it has good thermodynamic stability. Even after embedding 3–10 lithium atoms, its structure remains very stable. Therefore, this study demonstrates that embedding lithium ions in COF materials is completely feasible. The electronic properties of the material show that the insertion of Li atoms changes the inherent properties of the material, namely the electronic band structure and DOS. The material will transform into a conductor after being embedded with lithium ions. The reason for this change is due to the interaction between lithium atoms and organic groups in pure COF-IITI-0 structural units. Therefore, lithium-embedded porous materials can be used for lithium storage applications. Furthermore, Chen et al. [43] showed that the inserted thiophene group is also conducive to the adsorption and diffusion of Li^+^, the HOMO energy increases cause the band gap to narrow, the recombination energy decreases, and the charge redistribution is localized (Figure 5e,f). The small energy barrier of Li^+^ diffusion on BPT-COF also confirms that the inserted thiophene group can significantly reduce the energy barrier and improve lithium-ion conductivity (Figure 5g,h).

In summary, various COF materials based on carbonyl, triazine, imine, and other functional groups have been manufactured and utilized in LIBs. Most of the COF anodes are two-dimensional materials, which can shorten the transmission path and facilitate the rapid transfer of Li^+^. However, COF will undergo a thermally activated reversible reaction, leading to dissolution. Therefore, to improve the cycle stability of COF materials, they are usually compounded with graphene, carbon nanotubes, MXene, and other materials, or the structure is constructed with a double-active site. The heterostructure brought by two different materials can improve the lithium storage and charge transfer properties of the composite materials. At present, some research has been conducted on COF anodes in LIBs (Table 1). Through the analysis of the lithium storage mechanism, it can be seen that COF materials are very suitable for lithium ion embedding and can maintain a stable structure, which has broad application prospects in lithium-ion battery anode materials. 

## 3. Application of COF Materials in the Sodium-Ion Battery Anode

Although lithium-ion batteries have developed very rapidly, one of the most important obstacles to their development is the scarcity of lithium resources on the Earth. Therefore, the top priority of current research and development should be to develop other low-cost technologies to replace the energy storage related to lithium-ion batteries. In recent years, with the advent of the era of electric vehicles and smart grids, sodium-ion batteries (SIBs) have once again received widespread attention from the scientific research community due to their low price. At present, the key to the practical application of SIBs is to find high cycle stability and suitable commercial electrode materials for sodium-ion batteries [44].

At present, the most common application of COF materials in sodium-ion batteries is still the preparation of two-dimensional materials. Liu and his colleagues [45] continued to use the COF material system used in lithium-ion batteries to prepare millimeter-scale crystal covalent triazine frameworks (CTFs) with clear layered structures and peeled them off into several micrometer-scale 2DP sheets through micromechanical cracking and liquid ultrasound treatment, which exhibited good dispersibility in DMF solvents. Due to the strong conjugated porosity of 1D open channels arranged in crystal CTF and 2DP, they can provide fast and smooth diffusion paths for charge transfer and storage. These two modified composite materials provide extremely high reversible capacities of 225/262 and 67/119 mA h g^−1^ under 0.1 and 5.0 A g^−1^, respectively, and maintain 95% of their initial capacity of 1.0 A g^−1^ after 1200 cycles, outperforming most previously reported organic/polymer SIB anodes. Shehab et al. [46] synthesized microporous two-dimensional COF (Aza-COF) through the condensation reaction of hexaketocyclohexane with 1,2,4,5-benzenetetramine. The crystallinity and porosity of Aza-COF facilitates the fast and reversible access of Na^+^ and electrons to the dense phenazine redox active sites during electrochemical processes, and Na^+^ has the characteristics of reversible accommodation in Aza-COF porous channels. Therefore, electrodes based on Aza-COF exhibit quite high average specific capacity and cycle stability (550 mA h g^−1^ at 0.1 C), along with excellent energy and power density.

**Figure 6:**
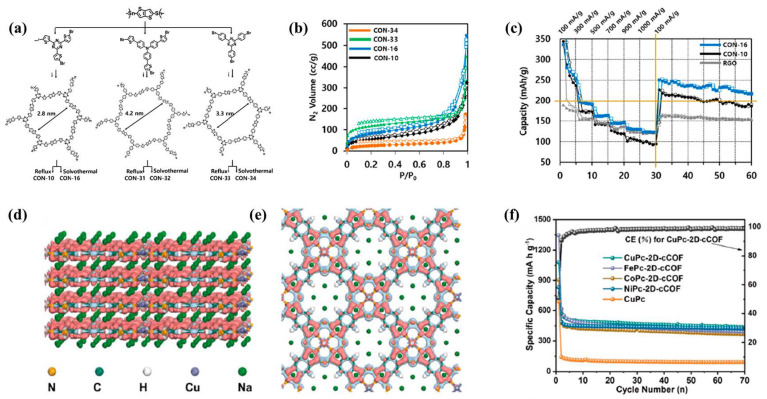
(**a**) General synthesis procedure and molecular structures of CONs with precursor monomers; “i” refers to 3-day reflux (CON-10, CON-31, and CON-33) or 3-day solvothermal treatment (CON-16, CON-32, and CON-34) in the presence of Pd0 in mesitylene at 170 °C, (**b**) N_2_ adsorption/desorption isotherms (empty/solid symbols and corresponding lines represent desorption/adsorption), (**c**) cyclic performance of CON electrodes under 100, 300, 500, 700, 900, and 1000 mA g^−1^ (and back to 100 mA g^−1^) compared with RGO. Empty symbols and corresponding lines represent the discharge capacities; solid symbols and corresponding lines represent the charge capacities of CON-10 and CON-16 compared with RGO [47]; differential charge density distribution along (**d**) a axis and (**e**) c axis after CuPc-2D-cCOF adsorbed Na ions, (**f**) electrochemical performance of as-synthetized MPc-2D-cCOFs and reference electrode cycle performance under 100 mA g^−1^ [49].

However, most 2D COFs show strong aggregation and readily form hydrolyzed species since they need to be synthesized via condensation reactions. Therefore, Kim et al. [47] prepared covalent organic nanosheet network polymers (CONs) via conventional reflux and solvothermal methods through Stille cross coupling, studying their structural dependence and energy storage performance. By controlling the specific surface area and self-assembly morphology of CONs, the planarity of the polymer main chain was enhanced, or by increasing its specific surface area while maintaining the main chain structure, the carrier conductivity was improved, and the storage capacity of sodium ions was increased. By adjusting the synthesis route of the reflux and solvothermal reaction, six types of CON anodes were obtained (Figure 6a,b). Comparison revealed that the electrode based on CON-16 exhibited the best performance, maintaining a reversible discharge capacity of about 250 mA h g^−1^ after 30 cycles under 100 mA g^−1^ (Figure 6c). Similarly, Hu et al. [11] used 2,6-diaminoanthraquinone (DAAQ) condensed with a hexachlorocyclotriphosphazene (HCCP) linker containing six substituent sites of -Cl, forming an ordered porous DAAQ-HCCP COF. The covalent bond formed between the nitrogen atom of DAAQ and the phosphorus atom of HCCP inhibits the dissolution of DAAQ in organic electrolyte, thus improving the stability of COF during charging and discharging. The ordered porous structure improves the diffusion rate of ions, and the condensation of redox-active quinones with linkers containing more substituents can increase the redox active portion in COFs, effectively increasing their specific capacity. The as-prepared COF showed a specific capacity of 88 mA h g^−1^ after 100 cycles at 100 mA g^−1^ and 72 mA h g^−1^ after 1000 cycles at 2000 mA g^−1^.

In addition, doping is also an important way to modify the anode material of SIBs. Xie et al. [48] introduced Sb^3+^ as an important catalyst for the formation of COF and further anchored it in the channel of COF through reduction (Sb@NGA-CMP). Ultrafine antimony nanoparticles are uniformly and densely encapsulated by the NGA-CMP framework. Nitrogen-containing groups provide a close electronic interaction between Sb nanoparticles and π-conjugated microporous polymers (CMPs), greatly accelerating the charge transfer along the COF framework and effectively accommodating the huge volume changes that are prone to occur with antimony in the electrochemical process, and this prevents aggregation. This unique anode design exhibits a high-rate performance of 223 mA h g^−1^ at 5 A g^−1^ and excellent sodium storage performance of 344 mA h g^−1^ after 5000 long cycles at 1 A g^−1^. Yang et al. [49] prepared fully π-conjugated and nitrogen-doped 2D COF (MPc-2D-cCOF) and used it as the anode material for SIBs. DFT calculation shows that, as shown in Figure 6d,e, after the adsorption of Na ions, the electron accumulation regions are mainly distributed around the aza-N and pyrrole-N atoms of CuPc-2D-cCOF, which further confirms the interaction between Na^+^ and the N atoms of the Pc macrocycle and the pyrrole-N conjugate part. At the same time, the electronic band structure of CuPc-2D-cCOF and CuPc is given by theoretical calculation. As a very important parameter of semiconductors, the electronic band structure can directly determine the conductivity of semiconductor materials. Therefore, the composite material exhibits excellent electrochemical performance (Figure 6f). On this basis, Haldar et al. [50] prepared three different mesoporous COFs (phenyl vs. tetrazine and bispyridine-tetrazine), which have almost the same highest occupied molecular orbital (HOMO) energy levels, but the lowest unoccupied molecular orbital (LUMO) energy levels are different. Compared with phenyl, the use of tetrazine/pyridine units reduces the LUMO energy level, making it easier for the former to accumulate electrons under external potential. This accumulated anti-bonding LUMO energy level provides a suitable driving force for the initially slow Na^+^ ions to enter the anode from the electrolyte.

**Figure 7:**
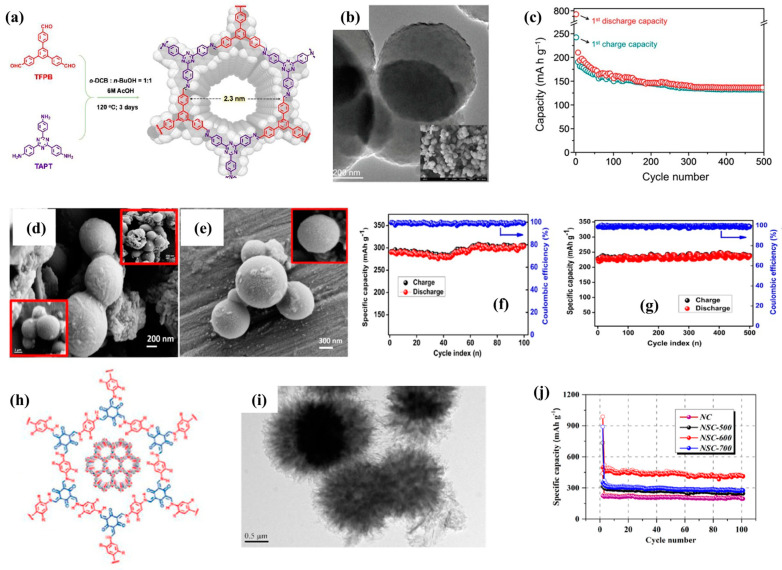
(**a**) Synthetic scheme of TFPB-TAPT COF, (**b**) TEM image of TFPB-TAPT COF, (**c**) long-term cycle performance of TFPB-TAPT COF [9]. (**d**,**e**) FE-SEM images of N-rich SC particles (inset: high magnification), (**f**) electrochemical performance of N-rich SC in capacity retention of SIBs as a function of cycle number, (**g**) steady-state cycling performance under 0.5 A g^−1^ [51]; (**h**) as-prepared COF, (**i**) TEM images of NSC-600, (**j**) cycling performances under 0.1 A g^−1^ [52].

Further, 3D COFs have also made some progress in the anode of SIBs in recent years. Patri et al. [9] synthesized a novel COF material with a C3-C3 symmetric topology, named TFPB-TAPT (Figure 7a,b). In SIBs, the average potential of the COF anode is 0.65V (vs. Na^+^/Na), and the Na^+^ ion storage capacity is mainly attributed to its open and ordered nanoporous framework, which provides reversible ion accommodation. Therefore, the initial reversible capacity of TFPB-TAPT COF is 246 mA h g^−1^ and can be effectively cycled up to 500 times (Figure 7c). Various carbon materials derived from three-dimensional COFs can also exhibit excellent performance. For example, Selvamani et al. [51] synthesized nitrogen-rich porous spherical carbon particles with large surface area through the facile pyrolysis of amorphous covalent organic frameworks (Figure 7d,e). The surface and edges of the carbon skeleton are uniformly grafted with nitrogen atoms, providing abundant active centers and exhibiting excellent electrochemical performance (Figure 7f,g), which can be attributed to its high level of nitrogen doping, large interlayer spacing, and specific surface area. In addition, compared to single doping, double doping can introduce more defects and accelerate ion diffusion, while the synergistic effect between double-doped atoms can significantly improve the electrochemical performance of the material. Zhang et al. [52] used COFs as precursors to prepare N/S co-doped sea-urchin-like porous carbons (NSCs) through a combination of carbonization and sulfurization (Figure 7h,i). The advantages of sea-urchin-like morphology and porous structure provide a stable internal structure, while dual doping endows it with rich defects, high electronic conductivity, and good capacitive behavior. Therefore, the composite material exhibits excellent sodium storage performance, with a capacity of 483.5 mA h g^−1^ after 100 cycles at 0.1 A g^−1^ (Figure 7j).

In summary, at present, because the mechanism of SIBs is similar to that of LIBs, most of the COF anode system of SIBs still adopts a structure similar to that of LIBs, and it has achieved relatively excellent performance (Table 1). In addition to the traditional 2D COF materials, 3D COFs are also beginning to be used in the anode of SIBs. By adjusting the COF structure, doping, and composite measures, the stability of the COF anode is significantly improved, the deactivation ratio is reduced, and the contribution capacity is significantly increased. Therefore, excellent cycling performance and rate performance can be achieved. It is worth noting that there is no activation phenomenon similar to that in LIBs during the electrochemical performance of the anode of SIBs. It is necessary to further determine the reason for this phenomenon through mechanism analysis.

## 4. Application of COF Materials in the Potassium-Ion Battery Anode

Potassium-ion batteries (PIBs) not only possess abundant resources but also have some other advantages, such as higher voltage plateau and energy density, as well as better conductivity of potassium electrolytes [53,54]. This means that PIBs are expected to replace LIBs as a new generation of battery energy storage system. The development of high-performance organic electrodes for PIBs has attracted attention due to their sustainability and low cost. However, electrolyte systems and anode materials that have been proven successful in high-performance LIBs have achieved relatively little success in PIBs.

**Figure 8:**
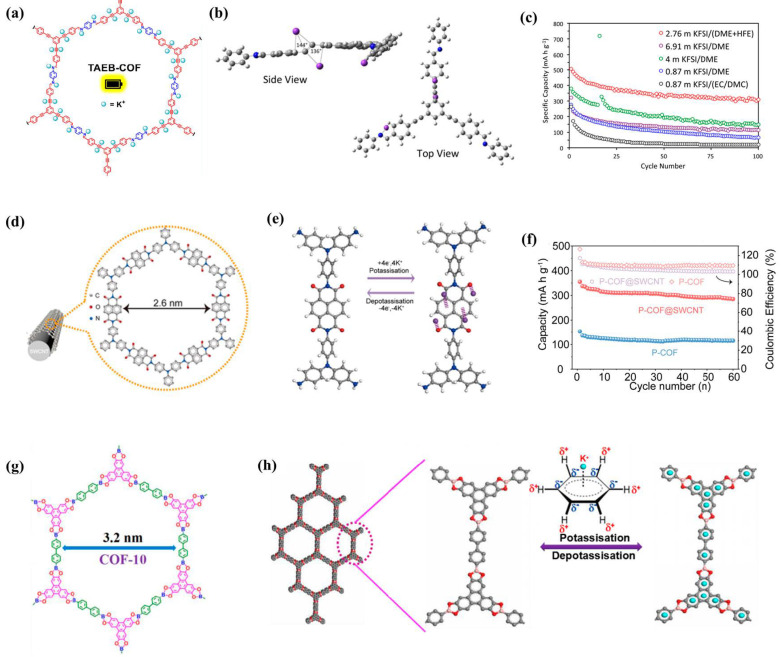
(**a**) Synthetic scheme of TAEB-COF, (**b**) visualization of optimized geometry (B4) of three K^+^ bound to radical anionic TAEB-PI. Deviation from the linear alkynyl bond (C-C-C) is shown in degrees, (**c**) charge capacities of TAEB-COF KIB coin cell fabricated with different electrolyte formulations at 50 mA g^−1^ [55]; (**d**) structural and morphological characterizations of P-COF@SWCNT, (**e**) schematic diagram of the proposed K+ storage mechanism (gray for carbon, red for oxygen, white for hydrogen, purple for potassium), (**f**) cycle performance of P-COF and P-COF@SWCNT at 0.1 A g^−1^ [57]; (**g**) structure of COF-10@CNT, (**h**) illustration of the proposed potassium storage mechanism [58].

Usually, the performance improvement in COF materials in PIBs is achieved through structural design or doping. Wolfson et al. [55] prepared various COF materials containing alkynyl functional groups (Figure 8a), and calculations showed that the presence of alkynyl groups can promote the binding of potassium ions through enthalpy and geometric driving force, leading to high reversible capacity (Figure 8b,c). Yang et al. [56] reported a fully aromatic-conjugated COF (FAC-Pc-COF) based on two-dimensional phthalocyanines, which has excellent physical and chemical stability and conductivity as high as 0.93–1.94 × 10^−4^ S cm^−1^, coupled with its highly ordered stable structure and N/O-rich skeleton, exhibiting high-performance K^+^ storage. After 100 cycles at 50 mA g^−1^, it exhibits a large reversible capacity of 424 mA h g^−1^. Especially under high-current conditions, due to its higher π-electron delocalization, which can improve electron transport, and the larger tunnel size, K^+^ ion transport can be enhanced, and the capacity retention rate approaches 100% after more than 10,000 cycles at 2000 mA g^−1^. Luo et al. [57] prepared in situ controlled polyimide COF on the surface of SWCNT (P-COF@SWCNT, Figure 8d). Through simple ball milling for stripping, more active site and various open channels are exposed, which shortens the diffusion path of ions in the electrolyte and prevents irreversible dissolution; ex situ FT-IR, XPS, Raman, and DFT calculations show that during the charge/discharge process, the C=O group can interact with K^+^, and the π-cation effect of the aromatic naphthalene ring provides an additional active site for K^+^ storage (Figure 8e); therefore, the P-COF@SWCNT anode exhibits an outstanding capacity of 438 mA h g^−1^ under 0.05 A g^−1^ (Figure 8f). Therefore, by introducing single-walled carbon nanotubes, a cross-linked conductive network is formed to accelerate the transfer of electrons from single-walled carbon nanotubes to P-COF, ensuring that the electroactive groups are fully utilized and can perform well, even at high current densities.

Therefore, using carbon nanotubes for composites is also a powerful method to improve performance. Chen et al. [58] successfully prepared a COF based on borate ester on the outer surface of carbon nanotubes (CNTs) by reasonably designing the organic condensation reaction (Figure 8g) and used it as the anode material for PIBs. The enhanced π-π stacking in fewer layers of COF-10@CNT COF can provide more exposed active sites, shorten the ion/electron diffusion distance, and enhance the insertion/removal kinetics of K^+^. In addition, the characterization of the potassium ion storage mechanism through XPS and Raman spectroscopy could be attributed to the π–K^+^ interaction between potassium ions and the COF-10 benzene ring (Figure 8h). Duan et al. [12] also explored the construction of COF anodes modified with carbon nanotubes and compared them with carboxylated carbon nanotubes (DAAQ-COF@CNT). Synthetic DAAQ-COF@CNTs have a large number of active sites, stable conductive skeleton, and suitable surface area of nanopores, which can provide high conductivity, shorten the ion/electron diffusion distance, and accelerate K^+^ diffusion. Comprehensive characterization via ex situ XPS, FT-IR, and DFT calculation indicates that the potassium storage mechanism of DAAQ-COF@CNT is the reversible insertion/extraction of K^+^ and its binding with C=O groups.

In 1864, Hugo Schiff reported the reaction of aniline with aldehydes to form imines, which were, therefore, named Schiff bases. In the reaction, amine carries out a nucleophilic attack on the carbonyl group, and after proton transfer, the semi-amine acetal formed by the secondary amine and hydroxyl group on the adjacent carbon atom is formed. The hydroxyl group of semi-amine acetal is protonated and dehydrated to form imine, which is deprotonated to form an imine with a carbon–nitrogen double bond. When molecules containing multiple amine groups react with molecules containing multiple carbonyl groups, Schiff base polymers can be prepared. Due to the presence of carbon nitrogen double bonds in Schiff bases, aromatic reactants can produce conjugated Schiff base polymers, while conjugated Schiff base polymers prepared using triamines (or trialdehydes) and dialdehydes (or diamines) can form highly cross-linked porous networks [59]. Polarographic studies have shown that organic electrolytes can undergo a reversible reaction at low potential, which inspired researchers to apply Schiff bases to anode materials for alkali metal ion batteries. Sun et al. [60] constructed a Schiff base COF/carbon nanotube (TP-COF/CNT) composite material for efficient PIBs anodes. By introducing carbon nanotubes, 1D hollow nanostructures were successfully constructed, exposing more active sites, thus giving the materials higher K^+^ transport dynamics and excellent conductivity. When used as an anode for PIBs, it has a high specific capacity and excellent electrochemical cycle stability. Furthermore, XPS, FT-IR, and DFT calculations show that the storage of K^+^ depends on the electroactive C=N group and π–K^+^ effect.

**Figure 9:**
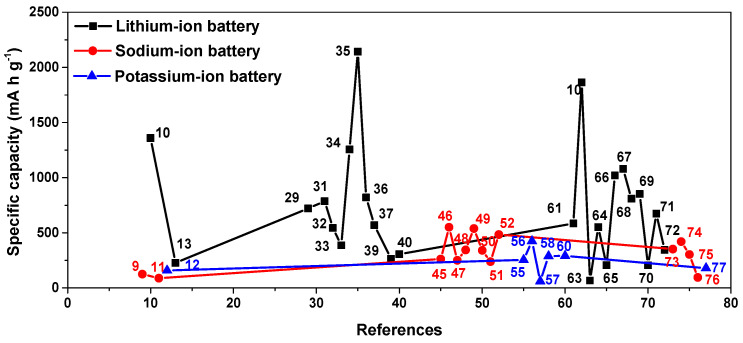
Performance comparison of COF materials in lithium-/sodium-/potassium-ion batteries.

In summary, the research on COF materials in potassium-ion batteries is currently relatively limited, but some progress has been made, Table 1 and Figure 9 shows the research progress and performance comparison of lithium-ion batteries, sodium-ion batteries, and potassium-ion batteries in recent years. The vast majority of materials have similar structures and, therefore, have similar functional groups and synergistic effects of π–K^+^. Similar to sodium-ion batteries, the main research direction of potassium-ion battery anodes is to increase the active sites and various open channels, increasing the efficient ion diffusion and preventing irreversible dissolution in the electrolyte. Meanwhile, its electrochemical process does not include the activation process.

**Table 1:** Performance of some COF materials in secondary-ion batteries.

Materials	Precursor COFs	Current Density (A g^−1^)	Cycle Number	Specific Capacity (mA h g^−1^)	Batteries	Ref.
TFPB-TAPT COF	TFPB-TAPT COF	0.03	500	125	SIBs	[9]
E-TFPB-COF/MnO_2_	TFPB-COF	0.1	300	1359	LIBs	[10]
DAAQ-HCCP COF	DAAQ-HCCP COF	0.1	100	88	SIBs	[11]
DAAQ-COF@CNT	DAAQ-COF	0.1	/	157.7	PIBs	[12]
TA-COF	TA-COF	5	2000	227	LIBs	[13]
CON	Trialdehyde and 3,5-diaminotriazole	0.1	1000	~720	LIBs	[29]
DAAQ-COF	DAAQ-COF	1	500	787	LIBs	[31]
PA-TA COF	PA-TA COF	1	400	543	LIBs	[32]
DCB-COF	DCB-COF	0.4	~500	387	LIBs	[33]
HAB-COF	HAB-COF	1	1100	1255	LIBs	[34]
FO@LZU1_50%_	COF-LZU1	0.1	300	2143	LIBs	[35]
COF-GQDs	COF-GQDs	0.1	300	~820	LIBs	[36]
COF@CNT-2	TP-DA-COF	0.1	100	570	LIBs	[37]
Tp-Ta-COF	Tp-Ta-COF	0.2	500	264	LIBs	[39]
Tp-Azo-COF	Tp-Azo-COF	1	3000	305.97	LIBs	[40]
Exfoliated 2DP	DCB-COF	0.1	/	262	SIBs	[45]
Aza-COF	Aza-COF	0.06	/	550	SIBs	[46]
CON-16	CON-16	0.1	30	~250	SIBs	[47]
Sb@NGA–CMP	NGA–CMP	1	5000	344	SIBs	[48]
MPc-2D-cCOFs	MPc-2D-cCOFs	0.05	/	538	SIBs	[49]
IISERP-COF18	bispyridine-tetrazine COF	1	/	340	SIBs	[50]
N-rich SC	PDA-COF	0.5	500	238	SIBs	[51]
NSC	TFP-PPD-COF	0.1	100	483.5	SIBs	[52]
TAEB-COF	TAEB-COF	0.05	300	254.0	PIBs	[55]
FAC-Pc-COF	QPP-FAC-Pc-COF	0.05	100	424	PIBs	[56]
P-COF@SWCNT	NTCDA- TAPA	0.1	282	60	PIBs	[57]
COF-10@CNT	COF-10	0.1	500	288	PIBs	[58]
TP-COF/CNTs	TP-COF	0.1	200	290	PIBs	[60]
Tf-TAPA@siloxene	Tf-TAPA	2	1000	583.6	LIBs	[61]
		8	1500	443.3		
Si@COF	TpPa	2	1000	1864	LIBs	[62]
DTP-ANDI-COF@CNTs	DTP-ANDI-COF	0.2	100	67	LIBs	[63]
CPOFs	POM	0.1	500	550.6	LIBs	[64]
DBA-COF 3	DBA	0.05	90	207	LIBs	[65]
Co-MOP@COF	COF-LZU1	0.1	150	1020	LIBs	[66]
Sn@COF-hollow	TAPB	0.1	100	1080	LIBs	[67]
H–Co_3_O_4_@CNBF	TAPT-DHNDA	0.2	100	808	LIBs	[68]
CeO_2_-NiO/NC	DHNDA-BTH	1	500	852	LIBs	[69]
NBCs	TAB-FPBA-COF	5	5000	205.5	LIBs	[70]
		10	5000	171.4		
NiO/NCF	TpPa	0.1	60	673.6	LIBs	[71]
NCM	DAB-Tp	5	400	345	LIBs	[72]
TQBQ-COF	TQBQ-COF	0.02	100	352.3	SIBs	[73]
DAAQ-COF	DAAQ-COF	0.1	100	420	SIBs	[74]
Carbonised COF	TAPB	0.1	/	303	SIBs	[75]
PICOF-1	Py-1P COF	0.069	175	95	SIBs	[76]
TPB-DMTP-COF	TPB-DMTP-COF	1	2000	179	PIBs	[77]

## 5. Conclusions

This paper reviews the application and research progress of the COF anode materials used in LIBs, SIBs, and PIBs in recent years. There are many studies on COF materials in the anode of LIBs, and their structures are also quite diverse. However, overall, the COF anodes of LIBs require a period of activation during the electrochemical cycling process to obtain stable capacity. Most of the anode materials in SIBs and PIBs follow the system of LIBs, but the research is still not perfect, which needs further analysis, and there is no activation process in both sodium-/potassium-ion batteries, so further research needs to be carried out in combination with the mechanism.

Multiple studies have shown that the electrochemical properties of COF anodes can be adjusted reasonably through different functional groups and porous structures. The current mainstream functional groups include C=C, C=O, C=N, C-N, N=N, π-conjugated benzene ring etc., all of which have correspondingly excellent performances, and through the introduction of multi-functional groups, more active sites can be introduced to increase the energy storage capacity. Therefore, the research on COF functional groups still needs to be further deepened.

Though COFs express promising advantages for metal-ion batteries, intrinsic challenges still exist, blocking the practical application of COFs as anode materials. (1) In order to achieve fast reaction kinetics, the electronic conductivity of COF materials still needs to be enhanced. Two ways could be considered to improve the conductivity. One is compounding COFs with electrically conductive guest materials, and the other is molecular structure modification, such as adjusting the direction of the conjugate π bond to increase intrinsic conductivity in all directions. (2) Potential dissolution in electrolytes needs to be further suppressed to alleviate capacity attenuation. Introducing special functional groups in organic ligand molecules, adding inhibitors to the electrolyte, or designing solid electrolytes may be effective ways to inhibit COF dissolution in electrolytes. (3) Low volumetric capacities still need to be improved due to the low density of COFs. It is necessary to explore novel morphologies and the structure of COF materials. (4) It is significant to investigate the storage mechanism of metal ions in COF materials by using advanced characterization techniques and theoretical calculations.

In conclusion, COF anodes have been relatively widely used in rechargeable batteries in recent years. As an organic anode, COF materials have the advantages of regular network structure, rich redox-active sites that can be modified, and fast particle transport paths, etc., which are bound to have a broader development prospect.

## Data Availability

The data presented in this study are available on request from the corresponding author.
